# Clinical and imaging findings in dogs with nerve root signature associated with cervical intervertebral disc herniation

**DOI:** 10.1111/jvim.16982

**Published:** 2024-01-12

**Authors:** Jordan Schachar, Alan Bocage, Nathan C. Nelson, Peter J. Early, Christopher L. Mariani, Natasha J. Olby, Karen R. Muñana

**Affiliations:** ^1^ Department of Clinical Sciences College of Veterinary Medicine, NC State University Raleigh, North Carolina 27606 USA; ^2^ Department of Molecular and Biomedical Sciences College of Veterinary Medicine, NC State University Raleigh, North Carolina 27606 USA; ^3^ Present address: Mount Laurel Animal Hospital Mount Laurel New Jersey USA; ^4^ Present address: Garden State Veterinary Specialists Tinton New Jersey USA

**Keywords:** cervical radiculopathy, intraforaminal disc herniation, magnetic resonance imaging, thoracic limb lameness

## Abstract

**Background:**

Intervertebral disc herniation (IVDH) is the most common spinal cord disease in dogs. Little information is available regarding the clinical presentation of nerve root signature (NRS) associated with cervical IVDH.

**Hypothesis/Objective:**

To detail the clinical and magnetic resonance imaging (MRI) findings in dogs with NRS associated with cervical IVDH.

**Animals:**

Forty‐seven client‐owned dogs presenting with thoracic limb NRS and MRI confirmed IVDH.

**Methods:**

Medical records from 2010 to 2020 were retrospectively reviewed for dogs that met inclusion criteria. Imaging studies were evaluated by 2 individuals to characterize location and severity of neural tissue compression.

**Results:**

Chondrodystrophoid dogs comprised the majority of the study cohort, with dachshund the most common breed (n = 10). Three‐quarters of dogs were ≥7 years of age. Interobserver agreement was moderate or good for all of the imaging variables evaluated. The C6‐C7 intervertebral disc space was significantly overrepresented (*P* = .01), comprising 32% (15/47) of the affected discs. However, 42% (20/47) of cases involved C2‐C3 though C4‐C5 disc sites. Disc material was more frequently located laterally compared to medially within the vertebral canal (*P* = .0005), and to be associated with compression of the nerve root at the level of the intervertebral foramen (*P* = .012).

**Conclusion/Clinical Importance:**

NRS is most commonly associated with lateralized or foraminal cervical disc herniations. It is most prevalent with C6‐C7 intervertebral disc involvement, suggesting that there might be unique anatomic factors that contribute to development of NRS at this site, but can be a clinical manifestation of IVDH occurring anywhere along the cervical spine.

**Abbreviations**
HASTE: half‐Fourier acquisition single‐shot turbo spin‐echo
IQR: interquartile range
IVDH: intervertebral disc herniation
MRI: magnetic resonance imaging
NRS: nerve root signature
PD: proton density
SD: standard deviation
STIR: short T1 inversion recovery

## 1 INTRODUCTION

Intervertebral disc herniation (IVDH) is a common neurological disorder of dogs, with involvement of the cervical spine reported in approximately 15% of cases.^1^, ^2^ Studies involving groups of dogs treated surgically for cervical IVDH describe one‐quarter to one‐half of affected dogs with nerve root signature (NRS),^3^, ^4^, ^5^ a clinical syndrome that presents as elevation of the limb in a non‐weight bearing position while standing, that is believed to be a manifestation of pain resulting from entrapment or irritation of the spinal nerve or nerve root.^6^

Nerve root signature is most commonly identified in association with IVDH and neoplastic conditions involving the nerve root, but can be seen with a variety of additional pathological etiologies including trauma, bony proliferative changes, inflammatory conditions, degenerative disease, and other neoplasia.^7^, ^8^ The pathophysiology of NRS is unknown, and likely multifactorial, but it is suspected to be caused by of a combination of compression, inflammation and vascular compromise to the sensitive nerve roots.^7^

Cervical radiculopathy is an analogous condition reported in humans in association with cervical disc disease. Affected patients describe pain that radiates in a myotomal distribution into the shoulder, arm, or digits with disc extrusions that affect the fifth through eighth cervical nerve roots.^9^ In a retrospective study of patients undergoing surgical treatment for cervical radiculopathy, 99.4% reported arm pain, with the C6 and C7 nerve roots most commonly involved.^10^ Similarly, it is generally accepted among veterinarians that cervical NRS in dogs is most often associated with involvement of nerve roots that originate from the C6 to T2 spinal cord segments of the cervical intumescence and innervate the muscles of the thoracic limb.^11^ However, the authors are not aware of any studies performed to objectively assess the clinical presentation of dogs with NRS. The goal of this study was to retrospectively evaluate the clinical and imaging findings of dogs presenting with NRS associated with cervical IVDH. We hypothesized that NRS is most common with disc herniations in the cervical intumescence, but can also be seen with disease involving the cranial cervical spine, and occurs more often when disc material extrudes or protrudes laterally in the vertebral canal or foramen, compressing a nerve root.

## 2 METHODS

### 2.1 Case selection

Medical records of cases admitted to NC State Veterinary Hospital from January 1, 2010 through June 1, 2020, were searched to identify dogs with a diagnosis of cervical IVDH that had clinical signs of NRS. Search terms included “intervertebral disc extrusion,” “intervertebral disc herniation,” “intervertebral disc disease,” “IVDD,” and “disc disease.” Inclusion criteria for the study were magnetic resonance imaging (MRI) confirmed cervical IVDH, and NRS identified on either physical examination or reported in the history obtained from the owner based on the presence of flexion of a forelimb to a non‐weight bearing position while standing. Dogs were excluded from the study if any indication of orthopedic disease of the affected limb was noted in the medical record, if cervical spinal pathology aside from IVDH was identified on imaging, if a diagnosis of hydrated nucleus pulposus extrusion or acute noncompressive nucleus pulposus extrusion was made on imaging, or if medical records or diagnostic imaging were incomplete. Data retrieved from medical records included age, breed, weight, sex, neuter status, and duration of clinical signs when available. Study dogs were categorized according to age, with older dogs designated as those ≥7 years of age. Similarly, dogs were classified as small or large breed based on weight, with small breed dogs ≤10 kg, and large breed dogs >10 kg.

### 2.2 Imaging review

Magnetic resonance imaging studies were independently reviewed using eUnity software (Client Outlook Inc, Waterloo, Ontario, Canada) by a veterinary neurology resident (JS) and a radiology resident (AB) who were blinded to dogs' clinical status. Images were evaluated within 3 broad categories, namely, IVDH location and laterality, spinal cord compression, and foraminal involvement, according to a rubric developed for the study (Table 1). Site of IVDH was recorded, and if multiple sites were present, the site suspected to be the cause of presenting clinical signs was agreed upon by reviewers based on indicators of acuity such as loss of signal on half‐Fourier acquisition single‐shot turbo spin‐echo (HASTE) sequence, signal intensity of extruded material, and presence of intramedullary T2 hyperintensity suggestive of edema. To evaluate the laterality of the extruded disc material, the vertebral canal was divided into quadrants, and the quadrant (left lateral, left medial, right medial, right lateral) with the largest volume of disc material was recorded. Spinal cord compression was graded from 0 to 3 using a modified spinal cord compression scale.^12^ Images with evidence of spinal cord compression (grade 3) were then further categorized based on the severity of compression (mild, moderate or severe), by calculating the ratio of the measured cross‐sectional area of the spinal cord at the point of maximum compression and the spinal cord cross sectional area at the adjacent cranial intervertebral disc space to determine the percent compression. Finally, any abnormalities in spinal cord intensity seen on T2‐weighted, STIR and post‐contrast images, if available, were recorded. Foraminal involvement was assessed as either present or absent. If present, the location of extruded disc material within the foramen was then assessed as proximal or distal. The spinal nerve was not consistently visualized because of varying degrees of compression. Therefore, impingement of foraminal height was assessed as an indirect marker of nerve root compression. The degree of reduced foraminal height was assessed on the affected side and compared to the contralateral intervertebral foramen, and categorized as either high or low degree of compression.

**TABLE 1:** Study rubric used to evaluate magnetic resonance images.

Category and variables	Sequences viewed	Scoring
Intervertebral disc extrusion		
Location	T2W, T1W sagittal, transverse	Record site of extrusion
Laterality of extrusion	T2W, PD transverse	Vertebral canal divided into 4 equal quadrants (left and right, medial and lateral) and quadrant with largest volume of disc material identified; foraminal material included in lateral quadrant
Spinal cord compression		
Grade of compression	T2W sagittal, transverse	Grade 0—no SAS compression
		Grade 1—partial SAS compression
		Grade 2—complete SAS compression
		Grade 3—spinal cord compression
Severity of spinal cord compression (for images with Grade 3 compression)	T2W, PD transverse	Ratio of cross‐sectional spinal cord area at (1) point of maximal compression and (2) adjacent, cranial disc space, to calculate % compression. Mild: <25% Moderate: 25%‐50% Severe: >50%
Spinal cord signal intensity	T2W, STIR sagittal	Hyperintense or hypointense as compared to normal spinal cord parenchyma
Foraminal involvement		
Material in the foramen	T2W, PD transverse	Yes/No
Location within the foramen (proximal vs distal)	T2W transverse	Straight line drawn from dorsal entrance of foramen adjacent to articular process to ventral exit of foramen adjacent to vertebral body; medial to line is proximal, lateral to line is distal
Foraminal nerve root compression (assessed as degree of reduced foraminal height	T2W, PD transverse	Ratio of average of 2 measures each of (1) line drawn perpendicular to the intervertebral foramen (articular facet to vertebral body) on affected side and (2) similar measurements on unaffected side; expressed as percent compression High compression: >50% reduction in foraminal height Low compression: ≤50% reduction in foraminal height

Abbreviations: PD, proton density; SAS, subarachnoid space; T1W, T1‐weighted; T2W, T2‐weighted.

### 2.3 Statistics

Continuous data are reported as mean and standard deviation (SD) if normally distributed, or as median and interquartile range (IQR) if there was a non‐normal distribution. The Shapiro‐Wilk test was used to test for normality. Categorical and ordinal data are reported as frequency of occurrence. For the MRI evaluation, agreement statistics for ordinal (non‐numeric) data were generated using the weighted version of Cohen's Kappa with quadratic weights. Quadratic weighting was selected to maximize the impact of large disagreements on the final agreement value while penalizing single‐category discrepancies less. The strength of agreement was interpreted as follows: values <0.20 were considered indicative of poor agreement, values 0.21‐0.40 were considered indicative of fair agreement, values 0.41‐0.60 were considered indicative of moderate agreement, values 0.61‐0.80 were considered indicative of good agreement, and values 0.81‐10.0 were considered indicative of very good or near perfect agreement. To report summary results for the imaging variables, the reviewers' percentage data for the categorical variables were averaged. To summarize values for degree of foraminal involvement from the 2 sets of ratings, the ratings were averaged (on a scale of none, low, high, designated by the numerical values 1 through 3, respectively) and rounded toward greater severity. A binomial test with a 1‐sided *P*‐value was used to compare the number of intervertebral disc extrusions cranial or caudal to C5, as well as the location of disc material within the vertebral canal (medial vs lateral) and the presence or absence of foraminal involvement, with a null hypothesis of an even distribution. To examine the distribution of IVDH across intervertebral discs, a multinomial test was used to detect significant departure from an even distribution across the 6 cervical intervertebral disc spaces. All statistical analyses were performed with available software (R version 4.2.1; R Project for Statistical Computing), using an established significance level of *P* < .05.

## 3 RESULTS

Fifty‐one dogs met initial inclusion criteria based on a medical record search of key terms, MRI confirmation of cervical IVDH and presence of NRS documented in history or on examination. Four dogs were excluded because of concurrent neurologic or orthopedic disease, with the remaining 47 dogs included in the study and imaging analysis. Twenty‐nine dogs (62%) were male, of which 27 were castrated, and 18 dogs (38%) were females, all spayed. Breeds included Dachshund (n = 10), mixed breed dog (n = 6), Beagle (n = 5), Labrador retriever (n = 4), Chihuahua (n = 3), Bichon Frise (n = 2), French bulldog (n = 2), Maltese (n = 2), Yorkshire terrier (n = 2), and 1 each of the following: American Cocker spaniel, Australian shepherd, Basset hound, Cavalier King Charles spaniel, Pomeranian, Poodle, Rat terrier, Rottweiler, Shih Tzu, Soft coated Wheaten terrier, and Staffordshire terrier. Twenty‐five of the 41 purebred dogs (61%) were of a breed considered to be chondrodystrophic with a genetic predisposition to IVDH based on the frequency of the FGF4‐retrogene mutation on chromosome 12.^13^ Mean age at time of presentation was 8.7 years (SD, 2.5 years), and median weight was 9.6 kg (IQR, 6.6‐19.2 kg). Twenty‐one (45%) dogs were classified as large breed dogs, weighing > 10 kg, whereas 26 (55%) dogs were considered small breed dogs (≤10 kg). Thirty‐six (77%) dogs were ≥7 years of age, and 11 dogs (23%) were <7 years of age.

Clinical signs of cervical hyperesthesia were present in all dogs. Additional abnormalities noted at the time of clinical evaluation included ataxia (n = 14), intermittent lameness (n = 7), tetraparesis (n = 2), and tetraplegia (n = 1). The dog with tetraplegia had a history of NRS before becoming recumbent. The location of the NRS (right vs left thoracic limb) was consistent with the laterality of the lesion identified on MRI in all dogs.

MRI of the cervical spine was performed using a 1.5 T (January 2010‐September 2018; Magnetom Symphony, Siemens Medical Solutions, Erlangen, Germany) or 3 T (October 2018‐June 2020; Skyra, Siemens Medical Solutions, Erlangen, Germany) scanner. Due to the study's retrospective nature, there was variation in the MRI sequences acquired among study participants. Acquired sequences included transverse and sagittal T1‐weighted pre‐ and post‐contrast, T2‐weighted, proton density (PD), short TI inversion recovery (STIR) and HASTE, with only T2‐weighted transverse and sagittal sequences performed in all studies.

The site of disc herniation was located at the C2‐C3, C3‐C4 or C4‐C5 intervertebral disc spaces in 20 (43%) dogs, and at the C5‐C6, C6‐C7 or C7‐T1 intervertebral disk spaces in the remaining 27 (57%) dogs, with no significant difference noted between these 2 locations along the cervical spine. The most commonly affected site was C6‐C7 (n = 15, 32%), followed by C5‐C6 (n = 9, 19%), C4‐C5 (n = 9, 19%), C3‐C4 (n = 7, 15%), C2‐C3 (n = 4, 9%), and C7‐T1 (n = 3, 6%). An unequal distribution of involved discs was identified among study dogs (*P* = .04), with C6‐C7 being overrepresented when compared to the other disc sites (*P* = .0095).

Interobserver agreement with respect to review of MRI studies was determined to be good for the variables of presence of spinal cord compression, severity of spinal cord compression, presence of foraminal involvement, and location of disc material within the foramen, with moderate interobserver agreement identified for the variables of laterality of disc material and degree of foraminal compression (Table 2).

**TABLE 2:** Summary of findings and interobserver agreement for imaging variables evaluated in dogs with nerve root signature associated with cervical intervertebral disc herniation.

Imaging variable	Observer 1	Observer 2	Interobserver agreement^a^
Spinal cord compression	n = 47	n = 47	0.73 (0.56‐0.92)
None (0)	6 (13%)	4 (8%)	
Partial subarachnoid compression (grade 1)	6 (13%)	6 (13%)	
Complete subarachnoid compression (grade 2)	4 (8%)	3 (6%)	
Spinal cord compression (grade 3)	31 (66%)	34 (73%)	
Severity of spinal cord compression	n = 31	n = 34	0.65 (0.46‐0.83)
Mild (<25% compression)	20 (65%)	22 (65%)	
Moderate (25%‐50% compression)	9 (29%)	11 (32%)	
Severe (>50% compression)	2 (6%)	1 (3%)	
Laterality	n = 47	n = 47	0.58 (0.34‐0.81)
Right lateral	14 (30%)	13 (28%)	
Right medial	9 (19%)	10 (21%)	
Left lateral	21 (45%)	15 (32%)	
Left medial	3 (6%)	9 (19%)	
Foraminal involvement	n = 47	n = 47	0.67 (0.46‐0.88)
Present	35 (74%)	28 (60%)	
Absent	12 (26%)	19 (40%)	
Location of disc within intervertebral foramen	n = 35	n = 28	0.65 (0.45‐0.85)
Proximal	32 (91%)	23 (82%)	
Distal	3 (9%)	5 (18%)	
Degree of foraminal compression	n = 35	n = 28	0.52 (0.34‐0.71)
Low (≤50% reduction in foraminal height)	12 (34%)	13 (46%)	
High (>50% reduction in foraminal height)	23 (66%)	15 (54%)	

^a^ Reported as Cohen's kappa, with confidence interval in parentheses. All values are statistically significant, with a *P*‐value <.001.

**FIGURE 1:**
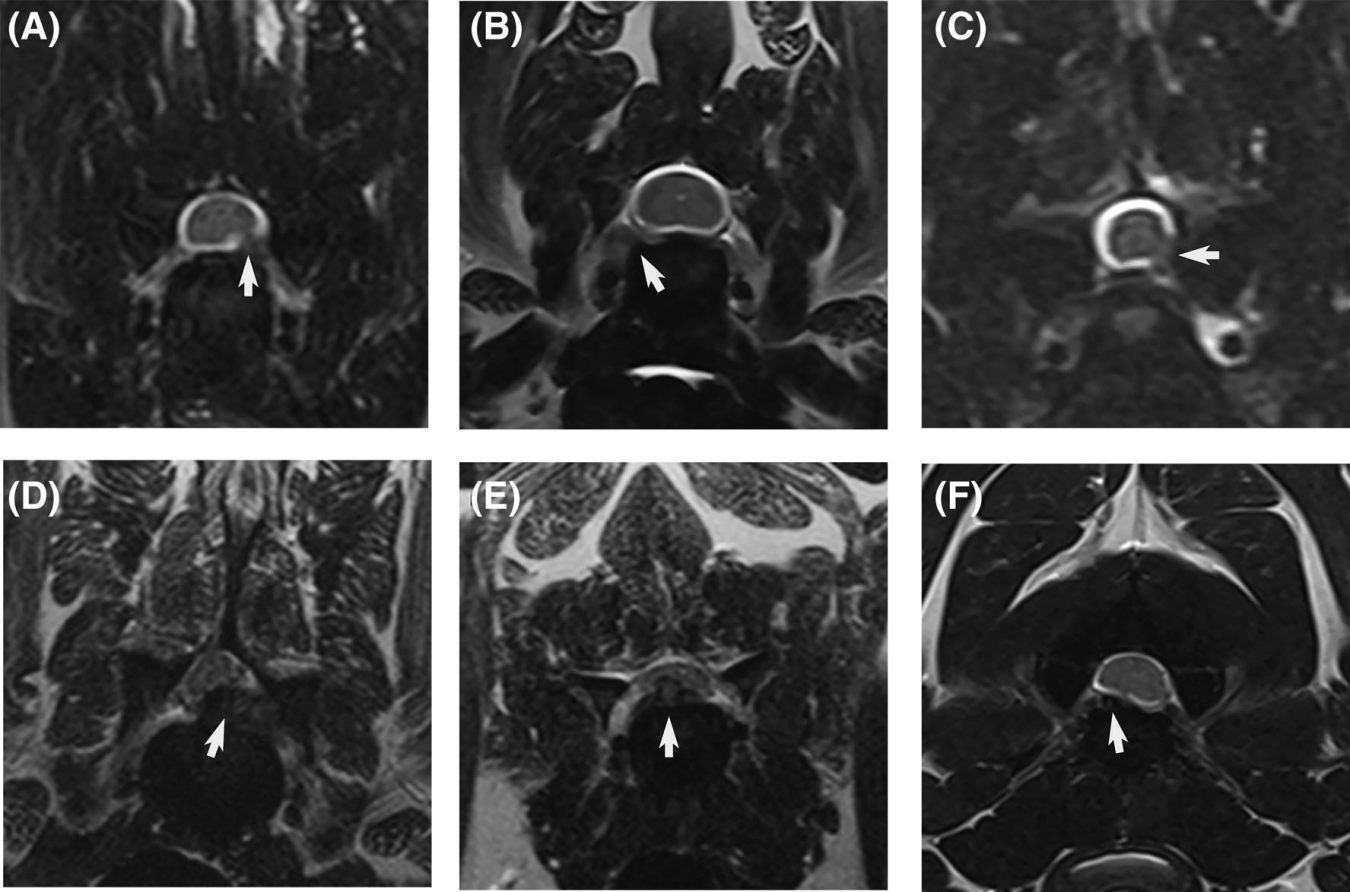
T2‐weighted transverse magnetic resonance images of the cervical spine of dogs with nerve root signature associated with intervertebral disc herniation demonstrating (A) C6‐C7 herniation resulting in mild compression of the left lateral aspect of the spinal cord and proximal foraminal compression (arrow); (B) C6‐C7 distal foraminal herniation (arrow); (C) C6‐C7 herniation with obliteration of the left ventrolateral subarachnoid space but no spinal cord compression, and proximal foraminal compression (arrow); (D) C6‐C7 herniation resulting in moderate compression of the spinal cord, most severe in the medial quadrant, and proximal foraminal compression (arrow); (E) C5‐C6 herniation with severe, spinal cord compression in the right medial quadrant (arrow); and (F) C2‐C3 herniation resulting in mild compression of the spinal cord, with the largest volume of extruded disc material noted in the right lateral quadrant (arrow).

Evidence of spinal cord or subarachnoid space compression was reported in 89% of dogs (42/47), with no compression noted in the remaining 11% (5/47) dogs (Figure 1). For dogs with spinal cord compression, the severity of spinal cord compression was assessed to be mild in 65% of cases, with the individual reviewers providing this assessment in 20/31 dogs and 22/34 dogs. Moderate compression was noted in 31% (9/31 and 11/34) of dogs, whereas severe compression was only noted in 3% (2/31 and 1/34) of dogs. One dog with severe compression was noted to have T2‐weighted hyperintensity of the spinal cord cranial and caudal to the compression. Other spinal cord changes included hydromyelia in 3 dogs and syringohydromyelia in 1 dog, all of which were located in the region of the spinal cord immediately cranial to the site of disc extrusion.

The predominant side of the disc herniation was roughly equally divided, with left‐sided herniations reported in 51% (24/47) of dogs, and right‐sided herniations reported in 49% (23/47). A significant difference was noted among study dogs with respect to the location of the disc material within the vertebral canal (*P* = .0005), with disc material 2.92 times more likely to be located laterally (95% CI, 0.61‐0.85) compared to medially (95% CI, 0.15‐0.40).

Study dogs were 2.0 times more likely to have disc material noted to be compressing the nerve at the level of the intervertebral foramen (95% CI, 0.54‐0.80) compared to no observable foraminal compression (95% CI, 0.20‐0.46; *P* = .012). Of the dogs with foraminal compression, 87% were determined to involve the proximal portion of the nerve root, with individual reviewers reporting this in 32/35 and 23/28 dogs, and distal foraminal compression was noted in 13% of dogs, with individual reporting frequencies of 3/35 and 5/25 (Figure 1). A high degree of foraminal compression, denoting >50% reduction in foraminal height, was identified most often, with average reporting frequency for high and low degree of compression of 60% (23/35 and 15/28) and 40% (12/35 and 13/28), respectively. Of the 33% of dogs that were judged to have no evidence of foraminal nerve root compression, the disc herniation was noted to involve the medial quadrant of the spinal canal more often than the lateral quadrant, with reviewers assessing the material to be located medially within the spinal canal in 63% of dogs (individual reviewer frequency of 7/12 and 13/19).

Ten dogs (21%) had evidence of IVDH at other locations throughout the cervical spine, but based on reviewer consensus of the degree of compression and the chronicity, these sites were judged to not be responsible for the clinical signs. Additionally, 1 dog had evidence of Chiari‐like malformation and associated syringohydromyelia, deemed an incidental finding.

## 4 DISCUSSION

This study evaluated the clinical and imaging findings in dogs with NRS associated with cervical IVDH, and confirmed that NRS is more likely to occur with disease in the caudal cervical spine with the C6‐C7 intervertebral disc site overrepresented compared to other disc sites. However, the study also documented that NRS can occur in association with IVDHs located anywhere along the cervical spine, with over 40% of study dogs having disease at intervertebral disc sites C2‐C3, C3‐C4 or C4‐C5, where the exiting nerves (the third through fifth cervical nerves, respectively) do not directly contribute to limb innervation in most animals. Finally, the study revealed that dogs with NRS are more likely to have herniated disc that is located laterally in the spinal canal or compromises the intervertebral foramen.

Nerve root signature is presumed to be caused by compression or inflammation of neural tissue, and is often described as being associated with nerve root rather than spinal cord involvement, and more specifically, nerve roots that contribute to innervation of the limbs.^7^, ^14^ The nerve roots are the most proximal portions of the spinal nerves as they emerge from the spinal cord. The 2 main nerve roots, the sensory and motor branches, fuse to form the peripheral nerves. Nerve roots are susceptible to injury because of their location within the rigid vertebral canal and intervertebral foramen that provides limited space to escape from mechanical forces and renders them vulnerable to compression.^15^ Nerve root signature is believed to be a clinical manifestation of a sharp or ‘shooting’ pain that travels down a limb, with animals assuming a flexed position of the limb in an attempt to alleviate the pain. Humans with cervical radiculopathy report that holding the affected arm above the head can help to alleviate pain, and this finding can be used to help support a diagnosis.^16^ However, our finding that NRS can be seen with disease affecting cranial cervical intervertebral disc sites and associated nerves suggests that this phenomenon is not solely because of involvement of the nerve roots that contribute to limb musculature innervation. Similar findings are reported in other studies. A report on intra‐foraminal and lateral cervical disc extrusions in dogs described referred pain to a thoracic limb as a clinical feature in 4 of 7 dogs, with involvement of intervertebral disc sites C3‐C4 in 2 of these dogs.^6^ The authors of this report attribute NRS to entrapment of a nerve root that contributes to innervation of the shoulder or extremity, and specify this to include the C4 through T2 nerve roots. However, the C4 nerve root is not considered part of the brachial plexus based on early anatomic studies.^17^ A second study of 13 dogs with cervical intervertebral foraminal disc extrusion reported 9 to have signs consistent with NRS, with involvement of intervertebral disc spaces C4‐C5 in 1 dog.^18^ It is possible that NRS in animals might be more dependent on the quality of pain or associated paresthesia, rather than the specific source of the pain; a “shooting” or “stabbing” pain perceived anywhere along the neck may prompt the animal to assume a posture with a limb lifted in an attempt to reduce pressure on affected nerve roots. Alternatively, IVDH could cause a regional inflammation that affects adjacent nerve roots. Experimental studies demonstrate an inflammatory response to extruded nucleus pulposus independent of compression, that can result in nerve root dysfunction.^19^, ^20^ There is a report of a dog with lumbar nerve root compression that presented with NRS of the opposite limb, which might suggest that the clinical manifestation of NRS can be quite variable with respect to lesion location and laterality.^21^ However, this was an isolated case report, and cases of opposing laterality were not identified in our study.

Lateral disc herniations would be more likely to affect, either by direct compression or adjacent inflammation, the nerve roots as they emerge from the spinal cord and vertebral canal. Our findings support the hypothesis that disease associated with the nerve root is more likely to lead to NRS. However, approximately one third of study dogs displayed no foraminal impingement. In these dogs, the disc herniation was noted to involve the medial quadrant of the vertebral canal more often than the lateral quadrant. NRS in these cases might be associated with more proximal compression or irritation of the nerve root within the canal, or a result of a local inflammatory response.

The predilection for NRS in the caudal cervical spine might be because of the increased likelihood of lateralized disease at more caudal disc sites attributed to inherent structural and anatomical characteristics. The most common site associated with NRS in this study was C6‐C7, followed by C5‐C6; this differs from the general site predilection for Hansen's type I cervical IVDH in dogs, which is most common at C2‐C3 with involvement progressively decreasing from C3‐C4 to C7‐T1.^1^, ^4^, ^5^, ^22^, ^23^, ^24^ This caudal cervical predilection is consistent with findings from studies assessing cervical radiculopathy in humans^25^ as well as foraminal intervertebral disc extrusion in dogs.^6^, ^18^ In addition, foraminal stenosis associated with cervical spondylomyelopathy is more common in the caudal cervical spine.^12^, ^26^

In humans, the height of the cervical intervertebral foramina (also known as the foraminal width) decreases caudally, with the smallest values identified at the C5‐C6 and C6‐C7 intervertebral disc spaces.^25^, ^27^ Additionally, a correlation has been identified between narrower disc spaces and smaller intervertebral foramina.^28^ A similar decrease in height of the intervertebral foramina in the caudal cervical spine is reported in Doberman pinschers, with a high incidence of foraminal stenosis identified in both clinically normal (69%) and affected (88%) dogs, and the site most commonly affected with both disc degeneration and foraminal stenosis being C6‐C7.^12^ This relative foraminal stenosis could predispose to a larger degree of nerve root compression in the presence of space occupying disease, such as IVDH, leading to increased incidence of NRS at caudal cervical locations.

Collapse of the intervertebral disc space is commonly noted in both humans and animals in association with extrusion or protrusion of disc material in IVDH. In addition to causing a decreased width of the disc itself, IVDH also causes narrowing of intervertebral foramina associated with diseased disc sites.^12^, ^29^ The disc space collapse and resulting foraminal narrowing can cause compression or irritation of the nerve roots as they exit the intervertebral foramen. Disc space collapse is a frequent cause of cervical radiculopathy in humans.^25^, ^29^, ^30^ Thus, disc space collapse, coupled with a greater degree of intervertebral foraminal stenosis, could predispose dogs to develop radiculopathy with IVDH located at more caudal sites in the cervical spine, resulting in a higher incidence of NRS. Disc space collapse was not a variable that was measured in this study, so the relationship between this and NRS could not be assessed.

This study did not attempt to differentiate between dogs with disc protrusions and disc extrusions, as surgical confirmation was not required for inclusion in the study. Studies on large breed dogs with thoracolumbar IVDH have identified imaging criteria that can assist in differentiating extrusions from protrusions, including the laterality of the disc material, the degree of intervertebral disc degeneration, involvement of a single rather than multiple disc sites and dispersion of disc material beyond the boundaries of the disc space.^31^ Of these criteria, only laterality was assessed in the present study, which demonstrated that dogs with NRS were significantly more likely to have disc material located laterally within the spinal canal. Although criteria for differentiating disc extrusions from protrusions have not been evaluated for the cervical spine in dogs, if the criteria established for thoracolumbar IVDH are predictive of disease in the cervical spine, this could suggest that the majority of study dogs had disc extrusions. The large number of chondrodystrophoid dogs in the study sample further supports a predilection for NRS with disc extrusions.

The study cohort was approximately equally divided by weight into small breed and large breed. This categorization was performed to determine if NRS was more common in small breed dogs that have been demonstrated to be prone to disc extrusion.^22^ Furthermore, approximately 1/3 of dogs were of breeds not considered chondrodystrophoid. Hence, although NRS appears to occur more often with disc extrusions, it is possible that it could also be seen with disc protrusions, possibly because of chemical rather than compressive irritation of the local nerve roots. A study involving surgical confirmation of disease would be needed to accurately determine the likelihood of NRS associated with cervical disc extrusions vs protrusions.

Over three‐quarters of dogs presenting with NRS in this study were over 7 years old. Although IVDH peaks in chondrodystrophic dogs between 3 and 7 years of age,^32^ cervical disc disease tends to affect older dogs.^3^, ^4^, ^5^, ^6^, ^18^ As dogs age, the prevalence of disc degeneration increases, resulting in a higher likelihood of disc collapse along the cervical spine. An increased prevalence of disc collapse could put older dogs at greater risk for developing NRS than younger dogs. However, a significant relationship between age and the presence of NRS was not identified in this study, and studies involving a larger sample size would be needed to evaluate this further.

Approximately one third of dogs with NRS in this study did not display any evidence of foraminal nerve root involvement on MRI, although lateralized disease occurred significantly more often than midline compression. This suggests that nerve root compression at any location, including within the spinal canal before the nerve exits the foramen, could be sufficient to cause NRS. However, it is also possible that observers underestimated the presence of foraminal nerve root compression because of poorly positioned MRI slices. To accurately evaluate foraminal nerve root compression, the MRI slices must be well aligned along the disc site and placed at an appropriate angle relative to the disc and intervertebral foramen. As imaging was not performed to specifically evaluate the foramen in this retrospective study, the position and angle of the images were not standardized and varied among cases. Some MRI slices were not accurately placed along the disc site or were placed at an inappropriate angle relative to the disc and intervertebral foramen. Additionally, the MRI studies utilized a general standard imaging protocol, consisting of T1‐weighted, T2‐weighted, STIR, and PD sequences. These sequences, because of their inherent technique as well as their application, could have contributed to underestimation of the presence of disc material, as well as variability in observer assessment of the degree and location of compression. Several additional sequence options can be considered in the future to better assess nerve root compression, foraminal stenosis, and impingement. Gradient echo images combined with magnetization transfer provides superior resolution and contrast when assessing foraminal stenosis.^26^ Angled sagittal MRI has been investigated in human medicine as a tool to facilitate evaluation of the intervertebral foramina.^33^ Finally, dynamic MRI sequences result in altered foraminal area in human studies.^34^ Dynamic studies as well as 3 dimensional isotropic MRI sequences of the cervical spine could be useful to allow more accurate assessment of foraminal disease because of their higher resolution, and ability to be reconstructed along the angle of the intervertebral foramen.

This study has several limitations, many owing to its retrospective nature. There was a lack of standardized imaging protocols and timing of imaging protocols relative to disease progression. Some dogs presenting with NRS were not imaged until clinical signs had progressed, which might have led to further disc extrusion in the interim. Imaging protocols were designed for general evaluation of the cervical spine and spinal cord and were not modified to focus on intervertebral foramina. Therefore, it is possible that the extent of foraminal involvement could have been underestimated depending on slices included or angle of slice obtained. Additionally, all MRI sequences were obtained in static cervical extension, and dynamic compression could have been missed. Extension of the cervical column decreases foraminal width in human studies,^35^ and positioning could lead to overestimation of foraminal compression in study dogs. Although all dogs had evidence of IVDH, a small number of cases displayed other abnormalities including other sites of IVDH and spinal cord compression, syringomyelia, and intraparenchymal changes. Although uncommon in the study cohort, it cannot be definitively excluded that these lesions contributed to development of NRS when present. These cases were included in final analysis as they accurately represent the clinical cases that present for evaluation. Finally, although reviewers were blinded to each case presentation, they were aware of the aims of the study, which might have influenced the evaluation of degrees of compression.

In conclusion, this study demonstrated that NRS can occur in association with IVDH at any disc site in the cervical spine, although it is significantly more common with involvement of the caudal cervical disc sites, particularly C6‐C7. In addition, NRS is more often identified with lateral disc herniations and those that result in foraminal compression, but it can also be present when these imaging findings are not apparent.

## CONFLICT OF INTEREST DECLARATION

Authors declare no conflict of interest.

## OFF‐LABEL ANTIMICROBIAL DECLARATION

Authors declare no off‐label use of antimicrobials.

## INSTITUTIONAL ANIMAL CARE AND USE COMMITTEE (IACUC) OR OTHER APPROVAL DECLARATION

Authors declare no IACUC or other approval was needed.

## HUMAN ETHICS APPROVAL DECLARATION

Authors declare human ethics approval was not needed for this study.
